# Editorial: Women in nanomedicine

**DOI:** 10.3389/fphar.2022.1122774

**Published:** 2023-01-05

**Authors:** Lay-Hong Chuah, Ju-Yen Fu, Sanko Nguyen, Manuela Banciu, Pratima R. Solanki, Hang Thu Ta

**Affiliations:** ^1^ School of Pharmacy, Monash University Malaysia, Bandar Sunway, Selangor, Malaysia; ^2^ Nutrition Unit, Malaysian Palm Oil Board, Bandar Baru Bangi, Selangor, Malaysia; ^3^ Department of Life Sciences and Health, Faculty of Health Sciences, Oslo Metropolitan University, Oslo, Norway; ^4^ Department of Molecular Biology and Biotechnology, Center of Systems Biology, Biodiversity, and Bioresources, Faculty of Biology and Geology, “Babes- Bolyai” University, Cluj-Napoca, Romania; ^5^ Special Centre for Nanoscience, Jawaharlal Nehru University, New Delhi, India; ^6^ School of Environment and Science, Griffith University, Brisbane, QLD, Australia

**Keywords:** nanomedicine, women in nanomedicine, nanoparticles, nanotheranostics, nanopharmaceuticals, nanotechnology

Over the years, women have made tremendous progress and contributions to education and the workplace alike. Despite various efforts to address equal opportunities, women are still largely underrepresented in many fields across the globe. The United Nations (UN) has listed gender equality as one of its Sustainable Development Goals (SDG) to address this global issue (United Nations, 2022).

Even though the number of women in Science, Technology, Engineering, Mathematics, and Medicine (STEMM) is growing, men continue to outnumber women, especially at the upper levels of these professions. Studies have shown that women comprise slightly less than a third of the STEMM research and development workforce. On top of that, women’s contributions are often overlooked, and they tend to experience isolation caused by a lack of access to women peers, role models, and mentors (AsiaRegional Bureau for Education, 2020). Another daunting fact reveals that women are often paid less than their male co-workers despite having similar expertise and experience (AsiaRegional Bureau for Education, 2020). This study has also shown that the rate of individuals leaving STEMM careers is disproportionately higher among women than men, especially working parents (AsiaRegional Bureau for Education, 2020).

Aligned with the UNSDG, more efforts need to be in place for us to achieve gender equality by 2030. This Research Topic presents the excellent work of women scientists in the field of nanomedicine research to create awareness and promote women who work in this area.

Nanomedicine has made significant technological advancements in cancer therapy, owing to its abilities to overcome the intrinsic shortcomings of conventional cancer therapies. A handful of nanoparticle-based medicines are clinically approved while more than 500 clinical trials are currently ongoing and indicated to treat all stages of cancer (Wang et al., 2022). Early development of cancer nanomedicine leveraged the enhanced permeability and retention (EPR) effect, which was later thought to be over-simplified. In De Silva et al., an in-depth investigation found using radiolabeled nanovesicles was an effective way to monitor and quantify the biodistribution of nanomedicine *via* real-time non-invasive imaging technique. This quantitative imaging technique was found to be positively associated with treatment outcome by addressing heterogeneity in tumor accumulation (Arrieta et al., 2014). While the EPR effect offers a predictive mechanism of nanomedicine distribution (Shi et al., 2017), it is often complicated by tumor heterogeneity, especially the tumor microenvironment (TME). In Negrea et al., functionalized long-circulating liposomes and polyethylene glycol-coated extracellular vesicles were synthesized to target tumor-associated macrophages and melanoma cells. Functionalized liposomes significantly altered protumor function of macrophages belonging to TME by sensitizing cancer cells to sequential administration of doxorubicin encapsulated in extracellular vesicles. The study is a proof of concept that integration of TME re-education in anticancer therapeutic approaches is a crucial factor in achieving maximum drug benefit. In line with the concept of synergism, combination strategies in anticancer therapy had proven success in generating positive clinical outcomes (Ayoub, 2021). Next-generation nanomedicine has expanded the options for combinatorial therapeutics. Liew et al. explored the use of liquid crystalline nanoparticles to co-encapsulate photosensitizers and chemotherapy for the treatment of pancreatic cancer. Despite distinctive physicochemical properties from both active compounds, the liquid crystalline nanoparticles synthesized were stable and homogenous with high entrapment efficiency. Incorporation of photodynamic therapy improves selectivity towards pancreatic ductal adenocarcinoma, which synergizes with gemcitabine to induce cell death.

The next-generation nanopharmaceuticals include highly intricate systems, often combining multiple strategies to promote drug delivery and improve therapeutic outcomes. In Attama et al., nanogels as target drug delivery systems in cancer therapy were reviewed. Simplified as hydrogels in nanoparticle form, nanogels are small enough to cross biological barriers, have high capacity to hold water without dissolving, and can be made stimuli-responsive to release drugs only under certain physical or physiological conditions (Soni et al., 2016). Attama et al. discussed how these nanogel features can be applied to overcome limitations of available cancer therapies. Another branch of next-generation nanopharmaceuticals is the “nanotheranostics” integrating therapeutic and diagnostic agents into single nanoplatforms. The multifunctionality of these drug delivery systems is particularly advantageous in cancer management allowing for simultaneous detection and tumor targeting, as well as post-treatment response monitoring (Chen et al., 2017). In a systematic review by How et al., nanotheranostics incorporating 5-fluorouracil, an old but still essential anti-cancer drug, was elucidated in terms of toxicity, anticancer efficacy, and imaging capability. The review highlighted the insufficient toxicological data on the nanosystems, necessitating more information to drive nanotheranostics from the preclinical to clinical stage. Another emerging nanomedicine is supramolecular mesoporous silica nanoparticles (MSNs), which offer distinct properties in terms of crystal structure, morphology-porosity, toxicity, and biological effects. The review by Mohamed et al. reports the use of MSNs as the next-generation drug delivery system, especially in designing oral controlled release dosage form. The history of MSNs, factors affecting morphology-porosity, advantages, safety aspects, and challenges faced in MSNs development were discussed in detail.

Although the development of nanopharmaceuticals has mainly been focused on cancer therapy and management, the use of nanocarriers to improve drug delivery is also applicable to other diseases and conditions. Bhandari et al. critically assessed the therapeutic benefits of nanoencapsulation of ocular drugs to improve the therapeutic outcome when treating conditions in the anterior segment of the eye. The authors concluded that the majority of the studies demonstrated some improved efficacy of drugs after encapsulation, although to variable degrees. The lack of *in vitro* - *in vivo* correlation in the studies was also discussed.

The increasing development of nanomedicine worldwide has led to the widespread investigation of its potential cytotoxic effects. A total of 70 articles assessing the cytotoxicity of chitosan nanoparticles were reviewed and reported by (Frigaard et al.). The review showed that chitosan nanoparticles express low cytotoxicity, but warned that new chitosan derivatives and compositions would require careful characterization and cytotoxicity assessments before being marketed. Safety and toxicity remain the major challenges in nanomedicine development.

The COVID-19 pandemic has accelerated the translation of nanomedicine use in humans, which has successfully broken the barriers of regulatory authorities. It is expected that more research into this area will be conducted in time to come, with greater funding supported by various stakeholders. The future of nanomedicine is promising, and there is an urgent need to address the gaps facing nanomedicine development in order to facilitate the advancement of this field.
